# The microbiomes of the eyelid and buccal area of patients with uveitic glaucoma

**DOI:** 10.1186/s12886-022-02395-x

**Published:** 2022-04-14

**Authors:** Jong Hoon Shin, Ji-Woong Lee, Su-Ho Lim, Byung Woo Yoon, Young Lee, Je Hyun Seo

**Affiliations:** 1 Department of Ophthalmology, Pusan National University Yangsan Hospital, Yangsan, Republic of Korea; 2 Department of Ophthalmology, Pusan National University Hospital, Busan, Republic of Korea; 3 Department of Ophthalmology, Daegu Veterans Health Service Medical Center, Daegu, Republic of Korea; 4 Division of Oncology, Department of Internal Medicine, Seoul Paik Hospital, Seoul, Republic of Korea; 5 Department of Internal Medicine, Chung-Ang University Gwangmyung Hospital, College of Medicine, Chung-Ang University, Seoul, Republic of Korea; 6 Veterans Health Service Medical Center, Veterans Medical Research Institute, Seoul, Republic of Korea

**Keywords:** Microbiome, Eyelid, Buccal, Uveitic glaucoma, Dysbiosis, *Lactococcus*

## Abstract

**Background:**

The microbiome could trigger inflammation leading to epigenetic changes and is involved in the pathophysiology of eye diseases; however, its effect on uveitic glaucoma (UG) has not been fully investigated. This study analysed the differences in eyelid and buccal microbiomes in patients with UG using next-generation sequencing.

**Methods:**

The eyelid and buccal specimens of 34 UG and 25 control patients were collected. The taxonomic composition of the microbiome was obtained via 16S ribosomal DNA sequencing. Diversity and differential gene expression analyses (DEG) determined taxon differences between the microbiomes of UG and control groups.

**Results:**

In both the eyelid and buccal microbiomes, alpha-diversity was lower in UG patients than controls, while beta-diversity in patients with UG was higher than in controls. DEG analysis of the eyelid microbiome revealed various taxa differences, including enrichment of *Paenibacillus* and *Dermacoccus* (*p*-value, 1.31e^−6^ and 1.55e^−7^, respectively) and depletion of *Morganella* and *Lactococcus* (*p*-value, 6.26e^−12^ and 2.55e^−6^, respectively) in patients with UG. In the buccal microbiome, taxa such as *Lactococcus* was significantly depleted (*p*-value, 1.31e^−17^), whereas *Faecalibacterium* was enriched in patients with UG (*p*-value, 6.12e^−8^).

**Conclusions:**

The eyelid and buccal microbiomes in patients with UG differ from controls, which raises concerns surrounding environmental influences on the pathogenesis of UG. The reduced *Lactococcus* in the eyelid and buccal area suggest that microbiota dysbiosis is associated with UG.

**Supplementary Information:**

The online version contains supplementary material available at 10.1186/s12886-022-02395-x.

## Background

The microbiome interacts with the human body and affects human health. Recent advances in next-generation sequencing (NGS) technology and bioinformatics tools have facilitated the study of the human microbiome [1], revealing that the epigenetic changes caused by the commensal microbiome contribute to the development of metabolic and systemic inflammatory diseases [2]. In addition, the emerging role of commensal microbiota may trigger immune responses and offer clues to the environmental origin of ocular inflammation such as uveitis [3–7]. Numerous studies in humans and animals have shown that uveitis is associated with gut microbiome changes [5, 8–16]. In a microbiome study for glaucoma on neurodegeneration, a higher rate of *Helicobacter pylori* infection was observed in patients with glaucoma than in non-glaucoma controls [17–19]. Previous studies on the oral microbiome of patients with glaucoma had identified specific microbiome biomarkers for OAG using machine learning [20] and showed an overall higher bacterial load, suggesting that the commensal microflora-associated immune response is mediated in glaucoma [21–23].

Uveitic glaucoma (UG) refers to a complex range of disorders defined by the coexistence of anterior uveitis with glaucomatous optic neuropathy, which encompasses several diverse clinical entities with different prognoses. In addition, our research group has previously observed differences in open-angle glaucoma (OAG) and UG microbiomes [24]. The microbiome may affect the pathogenesis of disease course-related factors of UG since uveitis and glaucoma are associated with the microbiome. To assess the microbiota factor in the UG pathogenesis, it is essential to compare the differences in microbiome diversity in UG compared with healthy people. Here, such knowledge should provide a basis for recognising microbiome biomarkers for the pathogenesis of UG. In this study, the eyelid margin microbiome was selected, including the lower conjunctival sac and meibomian gland, as the representative ocular microbiome because the ocular surface microbiota can be altered by environmental insults, and eyelid bacteria could enter the conjunctival area. The buccal microbiome, which is characterised as a gut microbiome, was selected as a representative of the non-ocular microbiome for uveitis in several studies [5, 6, 25, 26]. To date, few studies have focused on the microbiome of UG that characterises the components of uveitis and glaucoma. We hypothesise that there is a difference in diversity of the microbiome and specific taxa-related immune mechanisms in patients with UG compared to the control participants. Hence, this study aimed to analyse the microbiomes of the eyelid and buccal area and identify specific microbiomes associated with UG using NGS technology.

## Methods

### Participants

This multicentre study was conducted by the Veterans Health Service (VHS) Medical Center, Daegu Veterans Hospital, Pusan National University Hospital, and Pusan National University Yangsan Hospital. The study protocol was approved by the institutional review board of each centre. The study was performed according to the tenets outlined in the Declaration of Helsinki, and written informed consent was obtained from all participants prior to their inclusion in the study.

The researchers enrolled patients with UG who satisfied the inclusion criteria. UG is a complex range of disorders defined by the coexistence of anterior uveitis with glaucomatous optic neuropathy. Patients with UG had more than three previously observed instances of noninfectious anterior uveitis, regardless of the serological test and systemic disease. Glaucomatous optic neuropathy involves rim thinning, notching, retinal nerve fibre layer (RNFL) defects, and glaucomatous visual field defects. The control was defined as participants with a visual acuity of 20/40 or better and those without glaucomatous optic neuropathy, other retinal diseases, or a history of uveitis. Additionally, the control group did not have systemic inflammatory diseases, such as Behcet’s disease and ankylosing spondylitis. Furthermore, there was no history of steroid medication for eye treatment.

Participants with a history of ocular surgery other than uncomplicated cataract surgery, history of ocular trauma, or other diseases affecting the visual field (e.g., retinal vein occlusion, ischaemic optic neuropathy, etc.) were excluded. Moreover, participants who had food within 2 h before the swab, participants with a dental problem, contact lens wearers, tobacco smokers, participants with upper respiratory infection symptoms within the last 2 weeks, participants who were receiving oral antibiotics, oral probiotics, or topical antibiotic eye drops within 1 month were excluded from this study.

### Sample collection and DNA extraction

The protocol for collecting eyelid and buccal microbiomes samples was identical for all the centres. Eyelid margin swabs, including the lower conjunctival sac, were performed using 3 M™ Quick Swab (3 M Corporate, MN) with Alcaine (proparacaine HCl, Alcon, Geneva, Switzerland). The buccal swab was performed on the right and left buccal areas and under the tongue 4–6 times using the Gene kit (Daeilpharm Company, Seongnam, Korea). The samples were stored at − 20 °C before DNA extraction. Metagenomic DNA was extracted from swabs using GeneAll® Exgene™ Blood/Clinic/Cell SV mini kit (GeneAll, Seoul, Korea) according to the manufacturer’s instructions. The DNA quality control (DNA QC) criteria required 1) absorbance at 260 nm/280 nm ratio of 1.5–2.2 for DNA purity and 2) DNA concentration > 10 ng/µL using Trinean Dropsense 96 (Unchained Labs, Pleasanton, CA) and PicoGreen (Thermo Fisher Scientific, Waltham, MA), 3) presence of intact DNA band in 1^st^ PCR. If samples fail for DNA QC, an unreliable noise signal is generated, DNA sequencing was performed in samples with passed DNA QC.

### DNA Sequencing for targeting 16S ribosomal RNA

The DNA sequencing library targeting the V3 and V4 hypervariable regions of 16S ribosomal RNA was constructed according to the sequencing library preparation protocol (Illumina, San Diego, CA) in accordance with a previous study [27]. KAPA HiFi HotStart ReadyMix (Kapa Biosystems, Wilmington, MA) was utilised for PCR. The initial PCR was performed with 20 ng template DNA (DNA amount) using primers compatible with Illumina index and sequencing adapters (Table 1). Purification of the PCR product was performed with Agencourt AMPure XP system magnetic bead-based purification (Beckman Coulter Genomics, Brea, CA). After magnetic bead-based purification of PCR products, a second PCR was performed using primers from a Nextera XT Index Kit (Illumina) with a limited cycle. Subsequently, purified PCR products were visualised using gel electrophoresis and quantified with Qubit dsDNA HS Assay Kit (Thermo Fisher Scientific) on the Qubit 3.0 fluorometer [24]. The pooled samples were run on an Agilent 2100 bioanalyser (Agilent) for quality analysis before sequencing. Libraries were quantified with qPCR using a CFX96 Real-Time System (Bio-Rad, Hercules, CA). After normalisation, the prepared library was sequenced using the MiSeq system (Illumina) with 300 bp paired-end reads.

**Table 1:** Primers and adapters sequences for initial PCR

forward primer: 5’-TCGTCGGCAGCGTCAGATGTGTATAAGAGACAGCCTACGGGNGGCWGCAG-3’;
reverse primer: 5’-GTCTCGTGGGCTCGGAGATGTGTATAAGAGACAGGACTACHVGGGTATCTAATCC-3’

### Pre-processing of sequencing results

The adapter sequence was removed from the original paired-end reads using CutAdapt v1.11. Next, the merged reads were produced from the first processed paired-end reads using FLASH v1.2.11. Then, low-quality merged reads were filtered out according to the following criteria: the read contained two or more ambiguous nucleotides, the average quality score of the read would be < 20, or it’s length would be shorter than 300 bp after trimming low-quality bases. Finally, potential chimeric reads were removed using the previously published UCHIME v4.2.40 method [28].

### Calculation of operational taxonomic units

Pre-processed reads from each sample were used for calculating the number of operational taxonomic units (OTUs). The number of OTUs was determined by clustering the sequences from each sample with a 97% sequence identity cutoff using UPARSE and QIIME software (v.1.8.0) [29, 30]. Taxonomic abundance was counted with Ribosomal Database Project (RDP) Classifier v1.1, with a confidence threshold of 0.8 and derived from each sample's pre-processed reads [31]. The microbial composition was normalised among the samples by reading count using the value calculated by dividing the taxonomic abundance count by the number of pre-processed reads for each sample. Consensus sequences were clustered using cd-hit v4.6, with the following parameters applied: identify > 99% and coverage > 80%. Then, the consensus sequences were aligned to the databases from the National Centre for Biotechnology Information (NCBI) using the Mega BLAST algorithm. Finally, taxonomy profiling was performed for the assembled genome using NCBI taxonomy information.

### Diversity analysis and principal component analysis for microbial communities

Diversity analysis was performed in two ways: alpha-diversity, which is defined as the mean diversity of species within a sample, and beta-diversity, which is a measure of similarity or dissimilarity of two samples. The comparison of alpha-diversity was used in observing OTUs and the Shannon index. Additionally, the beta-diversity was compared using the weighted UniFrac distance among organism composition. Since there is a clinical significance of beta-diversity for group analysis, principal component analysis (PCA) was then performed using the result from beta-diversity with a multi-response permutation procedure (MRPP) [32]. Subgroup analysis was conducted according to age (cutoff age: 62 years old, the median age of the cohort). We calculated significant taxons using the Tag Count Comparison (TCC) project (http://bioconductor.org/), which provides functions for differential gene expression analysis (DEG) with normalisation and multi-group comparison [33]. In DEG, the volcano plot presents that the x-axis is the log2 of the fold change between UG and control, the y-axis represents the negative decade log of the significance (q-value).

### Statistical analyses

Data analyses were performed using R Statistical Package, Version 3.6.2 (R Foundation for Statistical Computing, Vienna, Austria) for statistical tests. A P-value of < 0.05 was considered statistically significant. The Wilcoxon test was performed to compare the beta diversity in each group. For multiple comparison tests, a false discovery rate (FDR) was controlled using the Benjamini–Hochberg step-up procedure [34, 35] for DEG analysis, and FDR < 0.01 was considered significant.

## Results

### The characteristics of participants and sequencing data

A total of 34 UG participants and 25 control participants were recruited from March 2019 to October 2020. The demographic information of the groups are presented in Table 2. There were significant differences between the two groups, including glaucoma-related variables and age. Subgroup analysis was performed to match age. In addition, the UG group had 6 participants with ankylosing spondylitis, 6 Posner-Schlossman syndrome, and 22 uveitis of unknown systemic disease. All buccal samples passed the DNA QC criteria; however, 14 eyelid samples from the UG group did not satisfy the DNA QC criteria. In this case, noisy signals may appear after NGS; therefore, sequencing was not performed. Merged sequences were generated from all the sequenced samples, resulting in an average yield of 168,307 ± 56,162 sequences/sample from the UG group and 192,814 ± 30,559 sequences/sample from the control group.

**Table 2:** Demographic and baseline characteristics of participants

**Variables**	**UG** ***N*** **= 34**	**Control participants** ***N*** **= 25**	***P*-value**
Age, years	54.41 ± 14.17	62.68 ± 1.73	0.002
Male / Female	24 / 10	18/ 7	0.903
BCVA, logMAR	0.30 ± 0.41	0.00 ± 0.20	0.0002
Hypertension, n (%)	4 (11.8)	5 (20.0)	0.384
Systemic inflammation disease Ankylosing spondylitis	6 (17.6)	-	-
Posner-Schlossman syndrome	6 (17.6)	-	
Not classified	22 (64.7)	-	
Glaucoma medication period, years	3.05 ± 3.03	-	-
Baseline IOP, mmHg	19.53 ± 8.99	13.48 ± 0.65	0.004
RNFL thickness, µm	69.53 ± 17.58	104.36 ± 5.62	< 0.0001
Visual field (MD), dB	-11.67 ± 10.43	0.08 ± 0.17	< 0.0001
Visual field (VFI), %	68.85 ± 33.68	99.84 ± 0.47	< 0.0001

*P*-values: Numeric data were analysed using the Chi-square test, and continuous data were analysed using independent t-tests

UG, uveitic glaucoma; BCVA, best-corrected visual acuity; logMAR, logarithm of the minimum angle of resolution; IOP, intraocular pressure; MD, mean deviation; RNFL, retinal nerve fibre layer; VFI, visual field index

### Comparison of alpha-diversity and beta-diversity

**Fig. 1:**
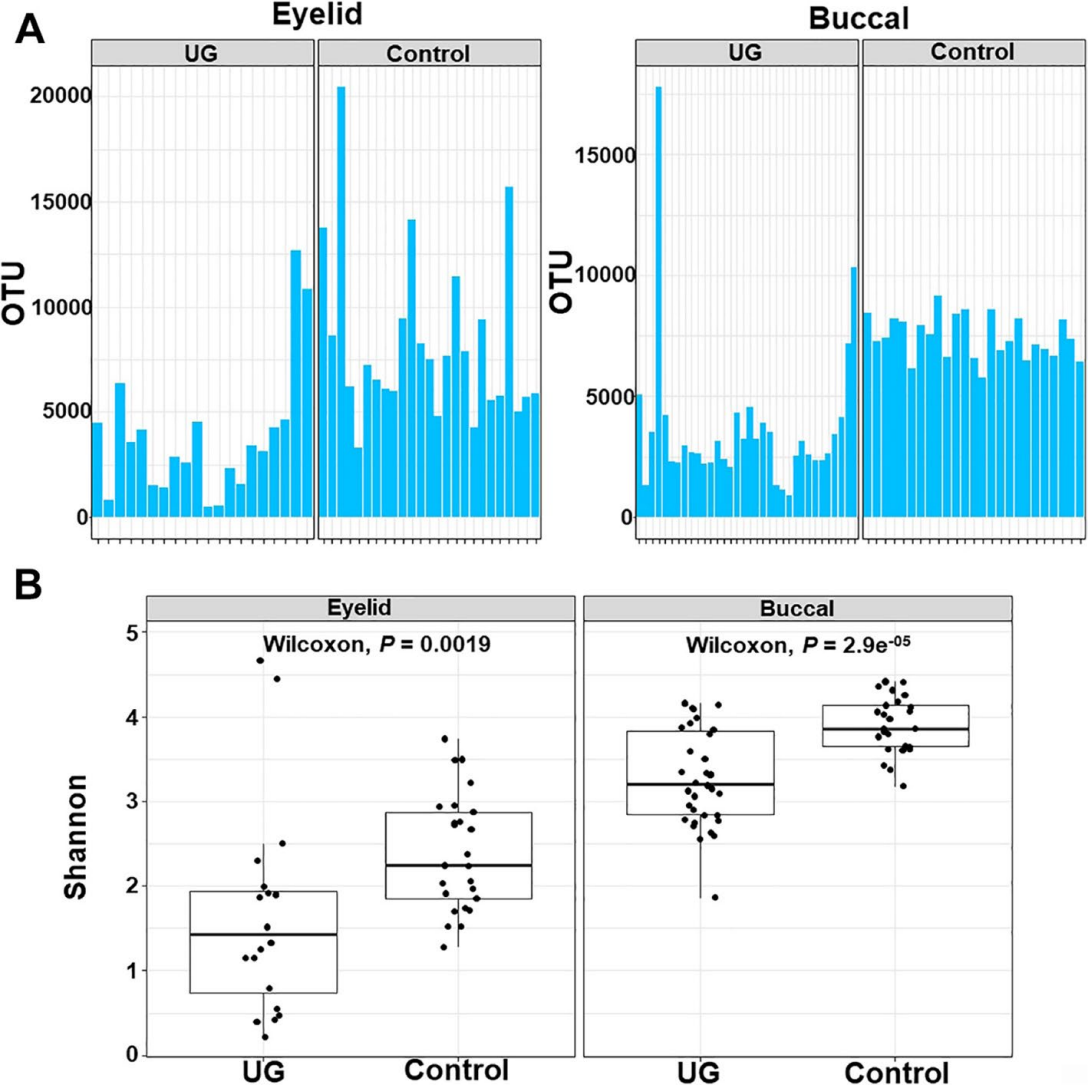
Relative composition of the bacterial community in eyelid and buccal microbiomes between patients with UG and control **A** It indicates alpha-diversity using OTU of enrolled subjects. **B** Alpha-diversity using the Shannon index was significantly different in eyelid and buccal microbiomes using the Wilcoxon test (*P* = 0.0019 and *P* = 2.9e^−5^, respectively).UG, uveitic glaucoma; OTU, operational taxonomic unit.

**Fig. 2:**
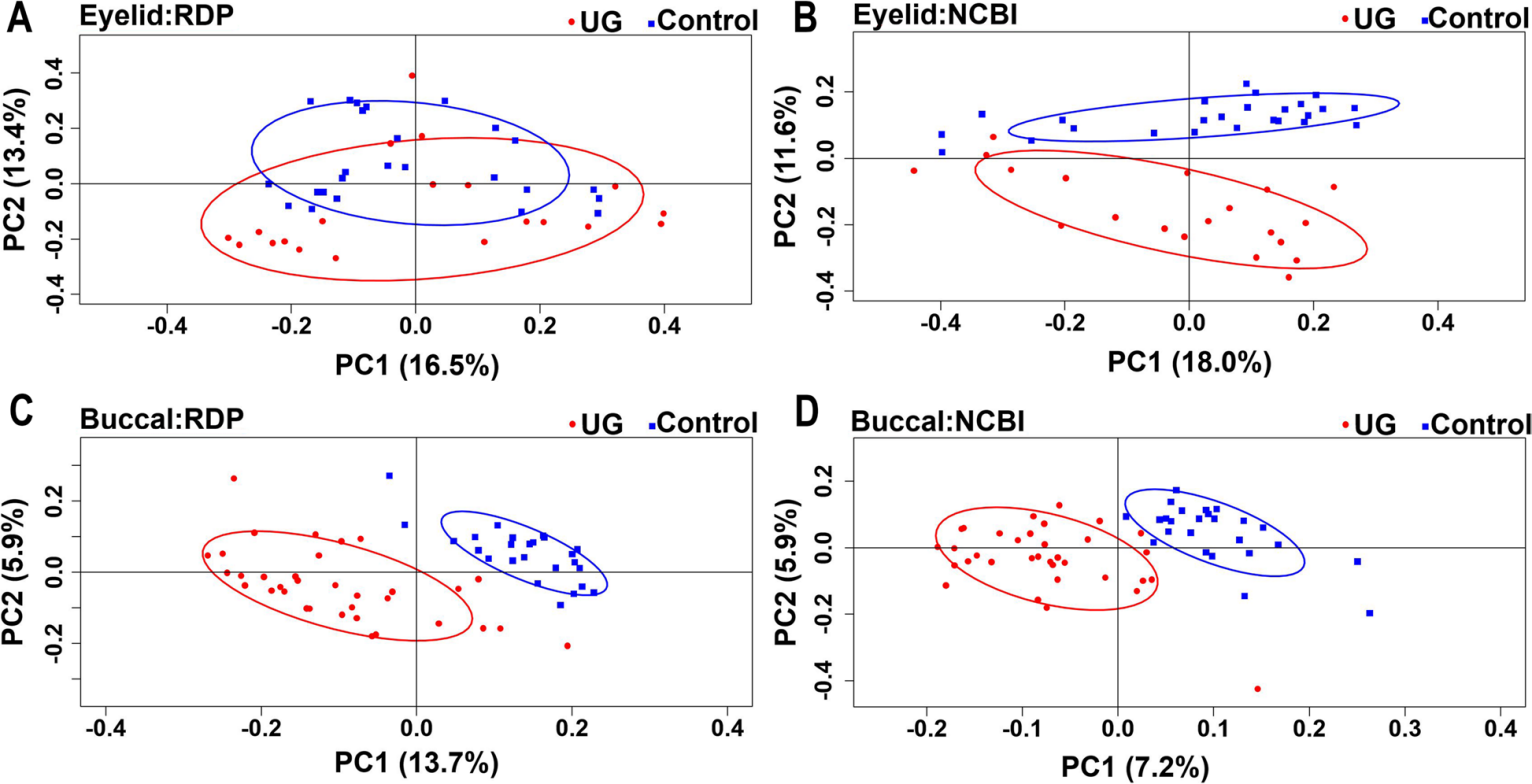
Differences in the microbiome of uveitic glaucoma using principal component analysis Using PCA of beta-diversity for the eyelid microbiome, UG and control clusters were explained differently. In addition, for the buccal microbiome, the UG cluster was different from the control cluster. The multi-response permutation procedure value of beta-diversity between UG and control was significantly different for the eyelid and buccal microbiomes using the RDP classifier (*P* < 0.001 and *P* < 0.001, respectively) and NCBI methods (*P* < 0.001 and *P* < 0.001, respectively). UG, uveitic glaucoma, RDP, Ribosomal Database Project; PCA, principal component analysis; NCBI, National Centre for Biotechnology Information using the Mega BLAST algorithm.

**Fig. 3:**
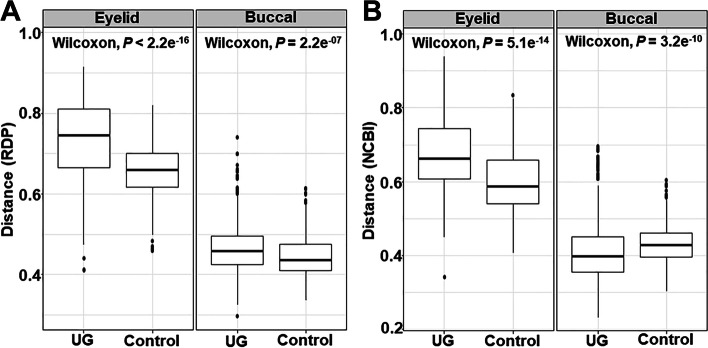
Beta-diversity in the microbiome of uveitic glaucoma Beta diversity (distance) was compared for each group using the Wilcoxon test. The distance was calculated from the taxonomy using the RDP classifier; the difference was observed in the UG group of the eyelid and buccal microbiomes (*P* < 2.2e^−16^ and *P* = 2.2e^−07^, respectively). In addition, a significant difference was observed for the eyelid microbiome (*P* = 5.1e^−14^) and buccal microbiome (*P* = 3.2e^−10^) based on the distance calculated from taxonomy using the NCBI database.UG, uveitic glaucoma, RDP, Ribosomal Database Project; NCBI, National Centre for Biotechnology Information

The comparisons of alpha-diversity using Shannon index between the UG and controls showed that alpha-diversity of the UG group was significantly lower than that of control groups in the eyelid and buccal microbiomes (*P* = 0.0019 and *P* = 2.9e^−05^, respectively; Fig. 1). Using PCA of beta-diversity for the eyelid and buccal microbiomes, the UG group differed from control clusters with statistical significance (Fig. 2) with the MRPP values RDP method (*P* < 0.001 and *P* < 0.001, respectively) and NCBI database (*P* < 0.001 and *P* < 0.001, respectively). The beta-diversity from the taxonomy using the RDP classifier was higher in the UG than in the control group of the eyelid and buccal microbiomes (*P* < 2.2e^−16^ and *P* = 2.2e^−07^, respectively; Fig. 3). In addition, significantly higher beta-diversity in the UG group was observed in the eyelid (*P* = 5.1e^−14^) from taxonomy using the NCBI database. In contrast, beta-diversity of the UG group in the buccal area was lower than that of the control group (*P* = 3.2e^−10^), respectively. Subgroup analysis (age > 62 years vs ≤ 62 years) showed that age did not affect the comparison results with the UG and control groups (Supplementary Figures S1 and S2).

### Taxon differences in the microbiome of UG compared with Controls

**Fig. 4:**
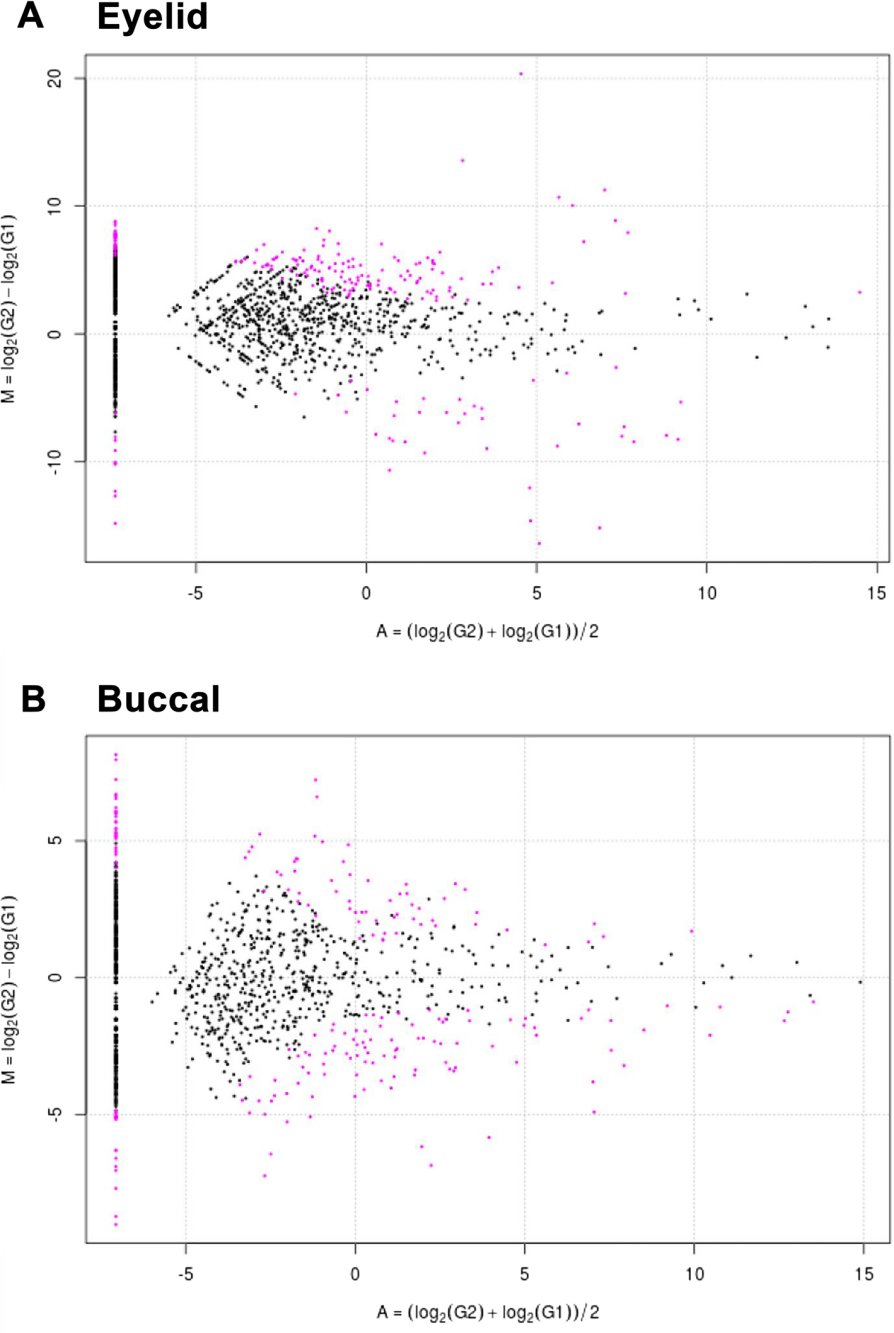
MA plots of the eyelid and buccal microbiome in UG patients In the eyelid microbiome, **A** MA plot of UG patients vs control participants (DEG number of the upper panel 274 vs lower panel 66, *P* < 0.05). In the buccal microbiome, **B** MA plot of UG patients vs control participants (DEG number of the upper panel 128 vs lower panel 147, *P* < 0.05).UG, uveitic glaucoma DEG, differential expression of genes

**Fig. 5:**
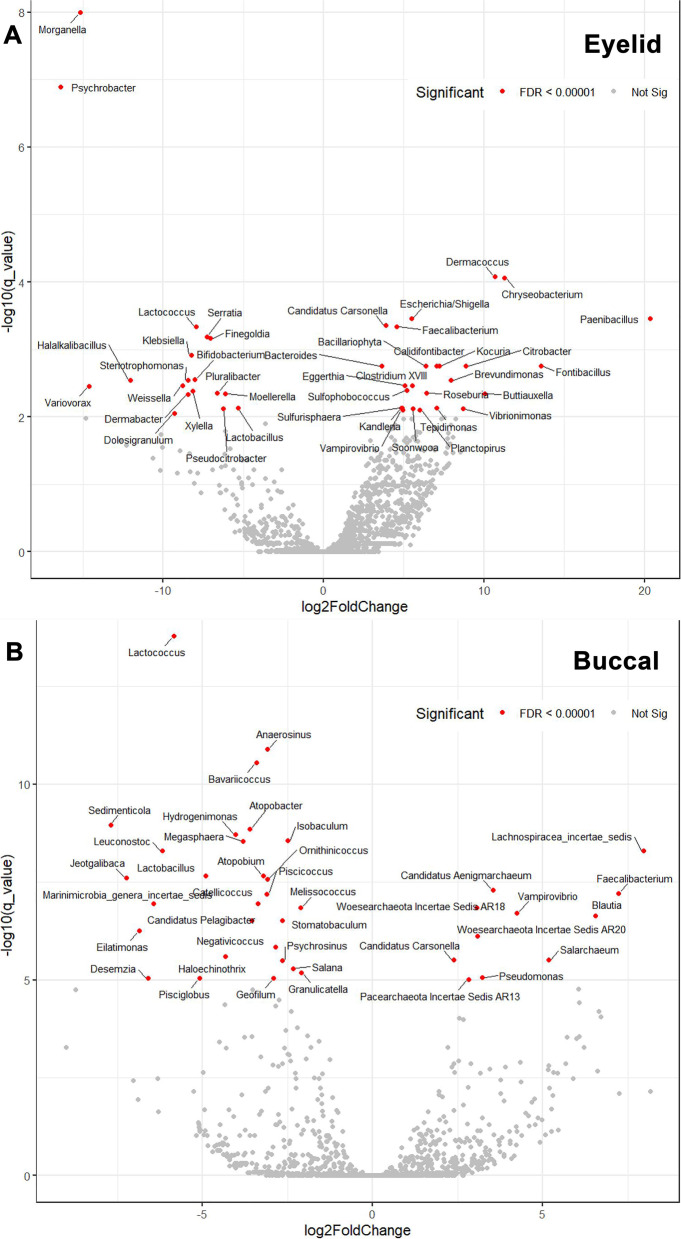
Volcano plots of the eyelid and buccal microbiome in UG patient comparison with control for DEG The x-axis is the log2 of the fold change between UG and control, the y-axis represents the negative decade log of the significance (*q-*value) **A** Volcano plot of the eyelid microbiome showed various taxa differences such as *Morganella*, *Psychrobacter*, and *Lactococcus* were depleted, whereas *Paenibacillus* and *Dermacoccus* were enriched in UG patients than in control participants. **B** Volcano plot of the buccal microbiome showed that taxa, such as *Lactococcus* and *Sedimenticola* were significantly depleted, whereas *Faecalibacterium, Lachnospiracea incertae sedis*, and *Pseudomonas* were enriched in UG patients than in control participants.UG, uveitic glaucoma; FDR, false discovery rate < 0.01 was significant (red indicates significance in FDR); DEG, differential expression of genes.

The MA plots revealed that the relative quantities of some species of the eyelid (upper panel 274 vs lower panel 66, *P* < 0.05) and buccal microbiomes (upper panel 128 vs lower panel 147, *P* < 0.05) were different in the UG group than in the control group (Fig. 4). The volcano plot from DEG analysis of the eyelid microbiome revealed various taxa differences, including enrichment of *Paenibacillus* and *Dermacoccus* (*p*-value = 1.31e^−6^, *p*-value = 1.55e^−7^, respectively) and depletion of *Morganella*, *Psychrobacter*, and *Lactococcus* (*p*-value = 6.26e^−12^, 1.27e^−7^, and 2.55 × 10^−6^, respectively) in participants with UG (Supplementary Table S1 and Fig. 5). In the buccal microbiome, taxa such as *Lactococcus* and *Sedimenticola* were significantly depleted in participants with UG (*p*-value = 1.31e^−17^ and 3.57e^−12^, respectively). In contrast, *Faecalibacterium*, *Lachnospiracea incertae sedis*, and *Pseudomonas* were enriched in participants with UG (*p*-value = 6.12e^−8^, 4.02e^−11^, and 2.41e^−7^, respectively) than in the control participants (Supplementary Table S2 and Fig. 5).

## Discussion

The commensal microflora of the human body maintains a symbiotic relationship with the host and contributes to the development of diseases [36]. Dysbiosis can induce or aggravate disease through toxic effects from direct invasion or epigenetic changes. Our study showed that the eyelid and buccal microbiomes of participants with UG differed from those of control participants, which was consistent in the subgroup analysis according to age. When compared with the controls group, alpha-diversity for UG was lower, whereas beta-diversity for UG was higher. These results suggested that UG has more diverse taxa with altered immunity than healthy populations. A taxa difference was observed for the eyelid and buccal microbiomes in the UG group compared with healthy controls. For clinical impact for this study, the genus of Lactococcus was significantly decreased in UG in both eyelid and buccal areas, which was related to anti-inflammatory strains [37]. In addition, several microbiome-related immune reactions were differently expressed in UG, although it is difficult to determine whether this phenomenon is the pathogenesis of UG or disease course-related factors such as anti-glaucoma medications or anti-inflammation medications.

Recent improvements in metagenomics analysis could lead to an in-depth characterisation of the microbiome across different diseases, as the microbiome is considered crucial in maintaining the homeostasis of the ocular surface [38]. Several conditions, such as dry eye syndrome and contact lens, are related to the ocular surface microbiome [38]. In this study, the eyelid microbiome for the UG group was identified as follows. The genus Paenibacillus was reported as one of the aetiologies of endophthalmitis following cataract surgery [39], while the genus Paenibacillus derived antimicrobials such as polymyxins have applications in medicine [40]. The genus Dermacoccus was abundant in the UG group in our study in contrast to the study of the microbiome for atopic dermatitis [41]. These results suggest that the inflammatory disease may differ depending on the activity and immune response. In addition, Morganella is gram-negative bacilli belonging to Enterobacteriaceae, known to be commensal in humans. However, Morganella could cause rare opportunistic infections of the eye, such as keratitis, endophthalmitis, and ophthalmia neonatorum [42, 43]. Psychrobacter was abundant in patients with fungal keratitis [44]; Lactococcus was reported to be associated with canaliculitis [45]. However, Morganella, Psychrobacter, and Lactococcus were depleted in the UG group in our study. These microbiome-related opportunistic infections in the eyelids of patients with UG were less common, which is thought to be related to anti-glaucoma medications with preservatives, noninfectious inflammation, anti-inflammatory, dysbiosis, and lifestyle. Considering our previous study on the preservative effect for eyelid microbiome [46], the preservative in anti-glaucoma medication is considered to affect decreased alpha-diversity in the UG group.

In the buccal area of the UG group, several microbiomes were significantly depleted and enriched as follows. The genus Lactococcus was significantly depleted in patients with UG, which is a probiotic bacterium that produces various bacteriocins and inhibits the bioactivity of oral pathogens [47]. Interestingly, a study on a synbiotic supplement for relieving anterior uveitis in Behcet’s disease using Lactobacillus and Bifidobacterium showed that lactic acid bacteria had an anti-inflammatory effect [48]. In terms of dysbiosis and symbiosis, it may be possible that UG occurs due to a decrease in strain-associated anti-inflammatory mechanisms; however, this hypothesis requires further study. The genus Sedimenticola is a genus of bacteria from the order of unclassified Gammaproteobacteria, and animal studies have been conducted regarding autoimmune uveitis [5]. In this study, Gammaproteobacteria tended to be inversely proportional to uveitis activity, and these results were consistent with our results [5].

A study on the gut microbiome has shown that the genus Faecalibacterium, which is known to be an anti-inflammatory organism, was enriched in healthy controls than in the uveitis group [11]. These results were not consistent with our findings, considering our gut specimen from the buccal area, although the previous study was from a faecal sample [11]. Moreover, according to a microbiome study on irritable bowel syndrome (IBS), the largest difference between patients with IBS and control participants was observed in the Lachnospiracea incertae sedis subnetwork [49]. This result supports our findings that UG is also an inflammatory disease. Pseudomonas has been reported to be abundant in patients with reactive arthritis and spondyloarthritis, which is consistent with our results [50]. These buccal microbiomes are related to pro-inflammation and anti-inflammation, which supports our findings that dysbiosis may be associated with anterior uveitis and glaucoma.

A recent study on the microbiome in UG compared with OAG had shown that Lactobacillus and Proteus was depleted, whereas Enterococcus was enriched in UG [24]. Combining these results with those of the present study suggests that lactic acid bacteria for UG decreased not only compared to healthy control but also compared to OAG. This dysbiosis might be related to the inflammatory component for UG. In addition, it should be considered as a potential factor affecting anti-inflammatory medications, such as steroids, anti-glaucoma medication, and anti-biotics during UG treatment. The high-fat and high-glycaemic index diet drives inflammation and angiogenesis in uveitis; therefore, further research is needed to determine whether it is a surrogate or pathological finding.

A major strength of our study is the inclusion of a relatively large number of patients with UG with metagenomic analysis and a multicentre base. In addition, both ocular and gut microbiomes were investigated, as the ocular microbiome may influence local inflammation in the eye, and the gut microbiome may influence local immune regulation. However, it was difficult to determine causality in this study, and there are several limitations to this study. First, the UG group was younger than the control group. According to a previous study on the effects of age on the microbiome [51], the Shannon index and abundance of microbiome were related to aging. In our study, beta-diversity was lower in the UG group than in the control group (older than UG), which is expected to offset the effect of age. Moreover, an age-based subgroup analysis was performed to minimise the age effect, which might have overcome this limitation. Second, this study was conducted as a multicentre study, and it could potentially be biased by the inclusion of people living in various locations in Korea. Third, patients with UG were enrolled with a wide range of phenotypes, such as active uveitis and uveitis-associated systemic disease. Furthermore, topical anti-inflammatory drugs, steroid medications, and anti-glaucoma medications may affect microbiome changes. Although this study focused on various UG, including Posner-Schlossman syndrome, a more well-organised subtype cohort needs to be created and analysed to address this problem.

## Conclusion

In conclusion, the microbiome of the eyelid and buccal area in patients with UG was different from that in control participants, which raised concerns surrounding environmental influences on the pathogenesis of UG. Although these microbiome differences may be secondary changes to related medications for UG management or pathophysiology related to UG itself, further study is necessary for answering these questions.

## Supplementary Information

**Additional file 1.** **Figure S1.** Analysis of subgroup of the eyelid and buccal microbiomes in patients with uveitic glaucoma (age >62 years) and control participants. Supplementary **Figure S2.** Analysis of subgroup of the eyelid and buccal microbiomes in patients with uveitic glaucoma (age ≤62 years) and control participants. 

**Additional file 2.** **Table S1**. Differential gene expression analysis of the eyelid microbiome in patients with uveitic glaucoma and control participants.

**Additional file 3. Table S2.** Differential gene expression analysis of the buccal microbiome in patients with uveitic glaucoma and control participants.

## Data Availability

The datasets generated and analysed during the current study are available from the corresponding author on reasonable request.

## Abbreviations

DEG: Differential expression of genes
DNA QC: DNA quality control
FDR: False discovery rate
IBS: Irritable bowel syndrome
MRPP: Multi-response permutation procedure
NCBI: National Centre for Biotechnology Information
NGS: Next-generation sequencing
OAG: Open-angle glaucoma
OTU: Operational taxonomic unit
PCA: Principal component analysis
RDP: Ribosomal Database Project
RNFL: Retinal nerve fibre layer
TCC: Tag Count Comparison
UG: Uveitic glaucoma
VHS: Veterans Health Service
