# Supplementary material for: The microbiomes of the eyelid and buccal area of patients with uveitic glaucoma
Source: BMC Ophthalmol. 2022 Apr 14;22:170. doi: 10.1186/s12886-022-02395-x (PMC9012020; doi:10.1186/s12886-022-02395-x)
Supplement: Supplementary file 1 — Additional file 1. Figure S1. Analysis of subgroup of the eyelid and buccal microbiomes in patients with uveitic glaucoma (age >62 years) and control participants. Supplementary Figure S2. Analysis of subgroup of the eyelid and buccal microbiomes in patients with uveitic glaucoma (age ≤62 years) and control participants. [file 12886_2022_2395_MOESM1_ESM.docx]

**Supplementary Figure S1. Analysis of subgroup of the eyelid and buccal microbiomes in uveitic glaucoma patients (age >62 years) and control participants.**

**
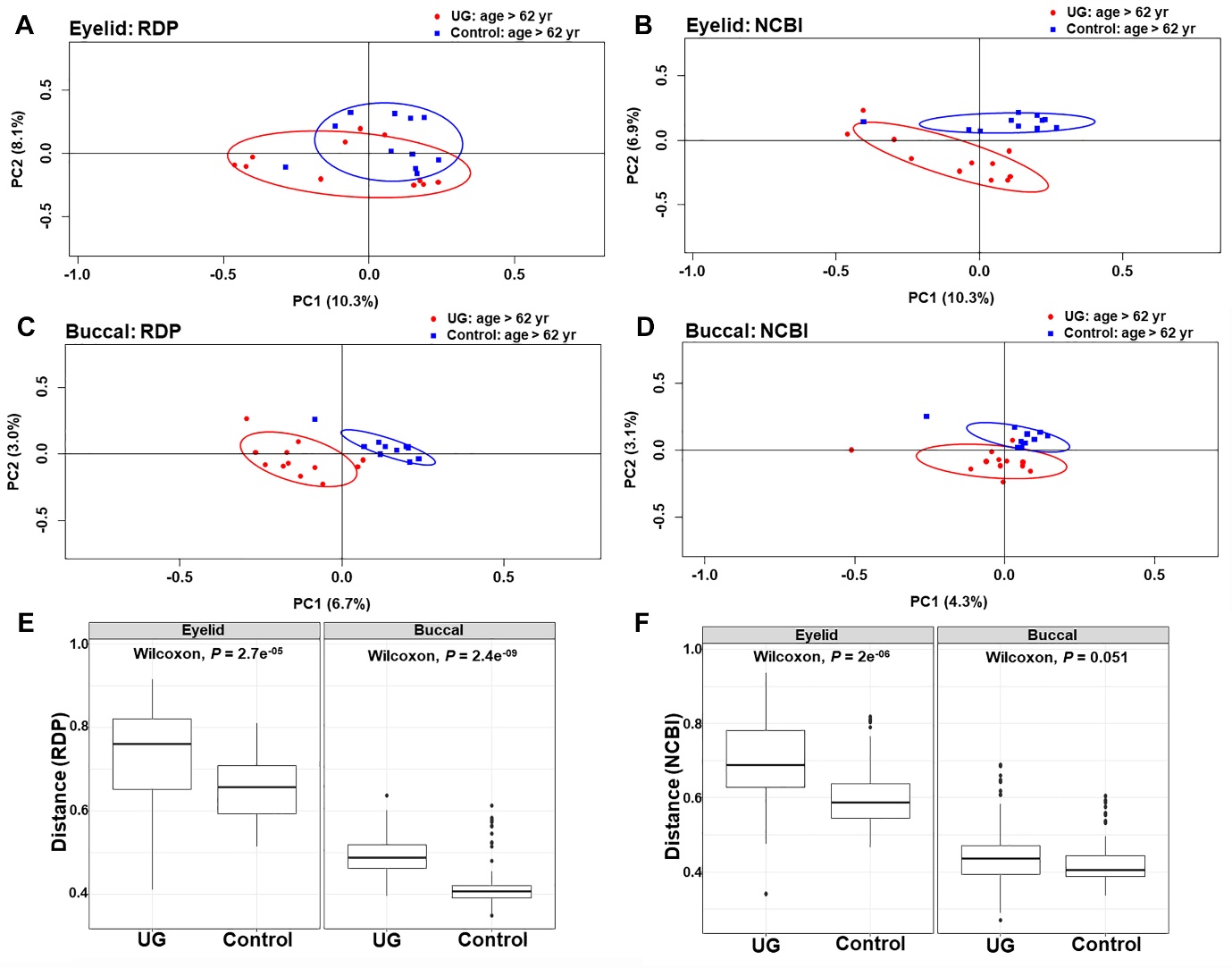
**

**A, C:** Beta-diversity analyzed from taxonomy using the RDP classifier in the eyelid and buccal microbiomes differed in the UG and control subgroups (age >62 years). PCA was used with the MRPP value (*P* = 0.0015 and *P* <0.001, respectively).

**B, D:** Beta-diversity analyzed from taxonomy using the NCBI database in the eyelid and buccal microbiomes differed between the UG and control subgroups (age >62 years). PCA was used with the MRPP value (*P* <0.001 and *P* <0.001, respectively).

**E:** The difference in distance calculated from the taxonomy using the RDP classifier was observed in the UG and control subgroups (age >62 years) of the eyelid and buccal microbiomes using the Wilcoxon test (*P* = 2.7e^−05^ and *P* = 2.4e^−09^, respectively).

**F:** The difference in distance calculated from the taxonomy using the NCBI database was observed in the UG and control subgroups (age >62 years) of the eyelid and buccal microbiomes using the Wilcoxon test (*P* = 2e^−06^ and *P* = 0.051, respectively).

UG, uveitic glaucoma; RDP, Ribosomal Database Project National Center for Biotechnology Information (NCBI) using the Mega BLAST algorithm, MRPP, multi-response permutation procedure**;** PCA, principal component analysis.

**Supplementary Figure S2. Analysis of subgroup of the eyelid and buccal microbiome in uveitic glaucoma patients (age ≤62 years) and control participants.**

**
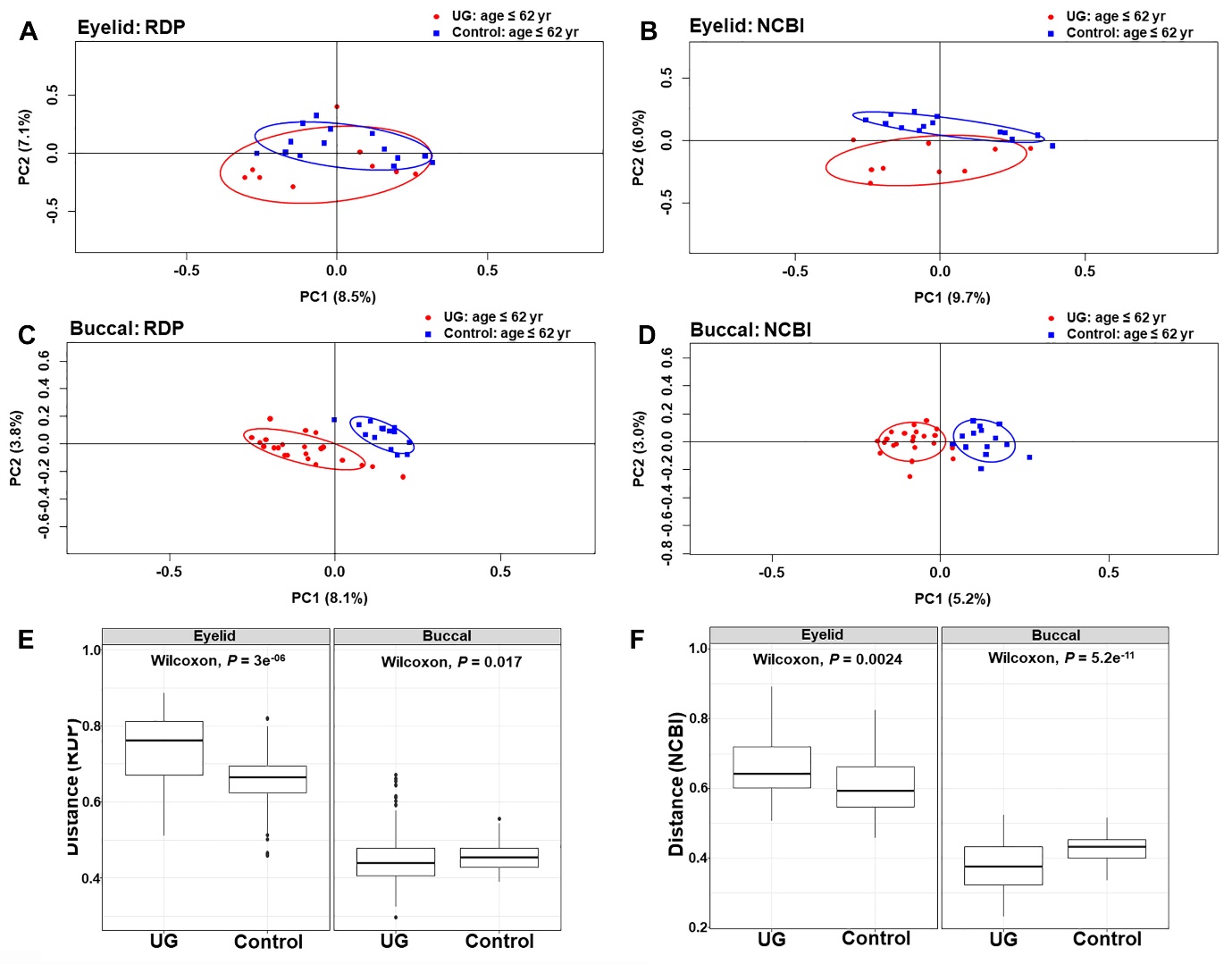
**

**A, C:** Beta-diversity analyzed from taxonomy using the RDP classifier in eyelid and buccal microbiomes differed in the UG and control subgroups (age ≤62 years old). PCA was used with the MRPP value (*P* = 0.0103 and *P* <0.001, respectively).

**B, D:** Beta-diversity analyzed from taxonomy using the NCBI database in eyelid and buccal microbiomes differed between the UG and control subgroups (age >62 years). PCA was used with the MRPP value (*P* = 0.015 and *P* <0.001, respectively).

**E:** The difference in distance calculated from the taxonomy using the RDP classifier was observed in the UG and control subgroups (age >62 years old) of the eyelid and buccal microbiomes using the Wilcoxon test (*P* = 3e^−06^ and *P* = 0.017, respectively).

**F:** The difference in distance calculated from the taxonomy using the NCBI database was observed in the UG and control subgroups (age >62 years) of the eyelid and buccal microbiomes using the Wilcoxon test (*P* = 0.0024 and *P* = 5.2e^−11^, respectively).

UG, uveitic glaucoma; RDP, Ribosomal Database Project; NCBI, National Center for Biotechnology Information using the Mega BLAST algorithm; MRPP, multi-response permutation procedure**;** PCA, principal component analysis.
